# The effect of cotton dust exposure as a long-term impact on lung function changes: A short narrative review

**DOI:** 10.5578/tt.20239614

**Published:** 2023-12-07

**Authors:** Naureen Akber ALİ, Noshaba AKBER, Adeel KHOJA

**Affiliations:** 1 School of Nursing and Midwifery, The Aga Khan University, Karachi, Pakistan; 2 Department of Sociology, Karachi University, Karachi, Pakistan; 3 Department of Medicine, Aga Khan University, Karachi, Pakistan

## INTRODUCTION

The effects of air pollution due to emissions from industries have detrimental impact on people’s health (1). Moreover, the potential health implication of these pollutants is significant to individuals working in these specific areas (1). Epidemiologic studies have identified that exposure to cotton dust is linked to a number of ailments, most of which are related to the respiratory system mainly lungs (2). Cotton dust contains varying sizes and types of particles that includes ground-up plant matter, fiber, bacteria, fungi, soil, pesticides, non-cotton matter, and other contaminants associated with lung function impairment (3). Different chronic pulmonary disorders, including asthma and chronic obstructive pulmonary disease are linked to rapid lung function decline (4). These illnesses are a major cause of morbidity and mortality and constitute a major burden on global health due to their rising prevalence (5,6). Evidence show that lung function is also a predictor of mortality, morbidity (7-9), physical and cognitive functioning (10). Due to the slow progression and chronic nature of decrease in lung function, changes in lung function over time are particularly interesting and provide options for prevention (11).

The findings of a cohort study have shown that cotton textile workers have a greater occurrence of respiratory symptoms and substantive chronic lung function loss (2,12). Moreover, increased pulmonary function losses may result from prolonged exposure to cotton dust (12). Functional loss has been determined to be influenced by exposure length, cumulative or mean dust concentration, previous levels of exposure, grade of cotton quality, and endotoxin levels (13,14).

Furthermore, observational studies have shown the impact of cotton dust exposure and cigarette smoking on the deterioration of pulmonary function to be additive (15,16). Smoking may worsen the negative effects of working in cotton yarn production as evident in longitudinal research (17). Although the long-term consequences of cotton dust exposure on the respiratory effects are well documented (17), some research studies have failed to identify effects of cotton dust on chronic effects of lung function (2). Studies have shown that bacterial endotoxins found in cotton dust may be the primary factors causing obstruction and inflammation of airways (18,19). Endotoxins are a part of the gram-negative bacterium’s outer membrane and are typically released from the bacteria by lysis and disperse throughout the entire airborne environment. High airborne levels are found in industrial settings such as cotton textile mills (20). A study has shown that endotoxin levels from environmental exposure are demonstrated to be marginally linked with cotton dust levels and do not differ noticeably between cotton mills (19). Therefore, uncertainty exists on the severity of lung function impairment following prolonged exposure to cotton dust, and it is significant to assess the impact of cotton dust exposure on change in lung function.

As the cotton industry is expanding in terms of production and employment, cotton dust-induced lung diseases are common (21). Considering the high burden of respiratory disease, it is significant to measure this occupational health hazard, where proper surveillance is not in place. Since lung function changes at work may serve as an indicator among cotton textile workers of subsequent or more severe loss. To our knowledge, there has not been a systematic review conducted on variations in lung function in relation to cotton dust exposure. Therefore, the purpose of this systematic review was to assess the impact of cotton dust exposure on cotton textile workers in relation to change in lung function over time and to determine the knowledge gap where more research is needed.

## MATERIALS and METHODS

We applied the methodology approach outlined by Levac and his coworkers, which was built upon prior studies by Arksey and O’Malley (22,23). This directed the steps, which included finding studies that were relevant in accordance with the research goal, choosing the suitable studies, and finally summarising

and presenting the results. This search guided the change in lung function among textile workers. The systematic review was conducted according to the steps of the preferred reporting items for systematic reviews and meta analyses (PRISMA) criteria.

## Information Sources and Search Strategy

The search plan and eligibility criteria were developed among the authors. The primary author performed the literature search to find relevant articles by looking at publications after 2000, in following online databases: Medline/PubMed, Web of Science, Science Direct, Google Scholar, and Cochrane Library. Using PubMed, the entire electronic search was streamlined before being repeated on other databases. The Additional file 1: Supplement 1 has a complete presentation of the electronic search strategy. Searches were made for additional suitable publications in the reference lists of all included studies and pertinent literature reviews.

## Inclusion Criteria

A broad search using the main search terms ‘cotton dust’ and ‘change in lung function’ was conducted to find relevant research studies. According to the authors’ assessments, this search was augmented with keywords for jobs such as cotton workers or cotton textile workers or cotton mill workers. Studies were searched based on the following inclusion criteria:

1) Study design: Prospective or longitudinal cohort studies with a ≥1 year follow-up time,
2) Exposure: Measurement of cotton dust exposure was performed through objective method in cotton textile mills,
3) Outcome: Long-term changes in the lung function indices; forced expiratory volume in the 1st second (FEV1),
4) Study subjects: Humans both male and females,
5) Language: English-language, full-length, original publications in peer-reviewed journals.

Studies were excluded based on;

1) Study design: Cross-sectional and follow-up time under one year, case reports, literature reviews and non-human studies,
2) Exposure: Other dust exposure or ‘mixed’ dust exposure unable to distinguish cotton dust,

The effect of cotton dust exposure as a long-term impact on lung function changes

3)
  Lack of exposure assessment,
4)
  Diagnostic studies of specific patient groups,
5) Sample size: Studies with less than 45 exposed subjects,
6) Lack of an estimate of association between lung function change and cotton dust exposure and
7)
  Lack of adjustment for smoking.

Varied cohorts were followed in various cycles and follow-up in different time periods (e.g., Shanghai Cotton Textile Study) (14) were reported based on the publications that were most relevant to our interests i.e., research aim and the exposure level (years of exposure). However, the studies focusing on the same population is briefly discussed in this review.

## Screening and Selection of Studies

All articles identified from the literature search were uploaded into the EndNote referencing software. Records were screened by two reviewers (NA & AK) independently. A pre-defined screening tool was designed, and a pilot testing was conducted. The selected studies were first screened by titles, followed by abstract, and finally full text screening to gradually exclude the studies that did not meet the eligibility criteria.

## Methodological Quality and Risk of Bias

Using several quality score systems is challenging (24), and previous criticism has been made on quality scoring system since it lacked validity (25), so we designed our own objectively based criteria mentioning important design elements instead of aggregate ratings. Therefore, quality of the studies was assessed and discussed on the following defined criteria concerning study design [longitudinal= 1

(all)], follow-up time (≥1 years= 1), response rate (>50%= 1), exposure measure (dust measurements= 1), and confounder control (all of smoking, sex, age and height= 1) as mentioned in Table 1.

## Study Selection

Studies that met the eligibility criteria from respective database were selected and evaluated. Following the elimination of duplicate publications (n= 589), there were 2580 pertinent studies (Figure 1), which were assessed based on title and abstract. This led to the exclusion of 2506 studies: 2226 based on their title and 280 based on their abstract. Following a full-text review of the remaining 74 papers, 69 were found to be irrelevant based on the research aim, study design, exposure size and lacked outcome assessment. Based on the eligibility criteria five studies were finalized for this review. The entire process of study screening and selection is exemplified in the PRISMA flow diagram in Figure 1.

## Characteristics of the Studies

Characteristics of the five included studies (2,17,19,26,27) are summarised in Table 2. All of the studies were prospective cohort studies with follow- up time varying between one and 25 years. Sample size ranged from 196 to 447 exposed individuals. Four studies had an external control group for comparison, (17,19,26,27) and one study had no control group (2). The mean age ranged from 35.3 to

60.8 years, and the cohorts originated from China (17,19,26,27) and Türkiye (2).

The included studies encompassed different years of exposure, i.e. 1) five years, 2) fifteen years, 3) twenty years, and 5) twenty-five years. In all studies, cotton dust was measured using a vertical elutriator (2,17,19,26,27). There were different outcome

**Table:** 

**Table 1.** Quality scores of the included studies								
**Author (year)**	**Research aim**	**Study design**	**Study population**	**Follow-up time**	**Response rate**	**Exposure measure**	**Confounder control**	**Total**
Wang, 2008	1	1	1	1	1	1	1	7
Christiani et al., 2001,	1	1	1	1	1	1	1	7
Wang et al., 2005	1	1	1	1	1	1	1	7
Hasan Kahraman et al., 2013	1	1	1	1	0	1	0	5
Shi et al., 2010, China	1	1	1	1	1	1	1	7

**Figure 1.** Flow diagram of study selection for the systematic review.

measures in all studies; however, we reported the outcome of our interest i.e., change in force expiratory volume in one second measured by spirometry with at least one-year follow-up.

## RESULTS

The results of the five included studies are summarised in Table 2. Four studies fitted generalized estimating equation (GEE) models (17,19,26,27) while one study performed multivariate linear regression (2) to determine factors associated for longitudinal changes in pulmonary function. Overall, three studies found an association between exposure and decline in lung

function (FEV1) (17,26,27) and two studies did not find any association between cotton dust exposure

and lung function (2,19).

Of the five studies on cotton workers, two studies were done by Wang et al. in 2008, 2005 (17,26). In one study, 408 cotton and 417 silk workers at textile factories in Shanghai were prospectively studied for

20 years, with baseline and follow-up surveys conducted at intervals of five years. Throughout the observation, the total follow-up rates were 74% or higher. Compared to silk workers, cotton workers

experienced larger, more frequent declines as well as significant chronic declines in lung function indices. Cotton dust exposure was related with a decline of 10 millilitre/year (mL/yr) in the five-year annualized FEV1 decline. Furthermore, there was a

1.5 mL/yr loss in annualised FEV1 reduction for every

10 mL drop or change in FEV1. An additional annualised drop of 7 mL was caused by smoking.

There was no obvious relationship between exposure and smoking. Over the course of the study, there was a higher correlation between the frequency of annualised reduction and drops for cotton workers compared to silk workers (17).

Another study prospectively witnessed 447 cotton textile workers and 472 silk textile controls, assessing the long-term effects of chronic cotton dust exposure on respiratory health, and the role of dust on lung function changes. The follow-up surveys were conducted in five-year intervals. At the last survey, 346 cotton and 342 silk workers from the previous survey were followed. This yielded follow-up rates of 84% in cotton and 77% in the silk groups. In the last survey, cotton dust in different areas were measured using vertical elutriators while inhalable air samplings

**Table:** 

**Table 2.** The effect of cotton dust exposure on the change in lung function over time, study results									
**S. No.**	**Author (year) (country)**	**Design, follow-up time**	**Study population, n**	**Mean age,**	**Sex**	**Cotton dust exposure measurement**	** Outcome: Lung function change measurement **	**Covariates accounted**	**Findings**
1	Xiaorong Wang,	Prospective	408 cotton	37.1	190	Airborne cotton	Spirometry:	Age, height,	Exposure to cotton dust was
	2008, Shanghai,	cohort study,	workers and	(10.4)	(46.6) M	dust were	FEV1	male, smoking,	associated with a 10 mL/year
	China	20 years	417 silk			measured using		year of retire-	decrement in five-year
		follow-up	workers from			a vertical elutri-		ment, exposure	annualized FEV1 decline. In
			Shanghai			ator		to endotoxin,	addition, every 10 mL in
			textile mills					exposure to dust,	DFEV1 drop was associated
								and change in	with an additional 1.5 mL/
								FEV1, 10 mL	year loss in annualized FEV1
									decline. The association
									between the frequency of
									drops and annualized decline
									was stronger for cotton
									workers than for silk workers
									over the entire study period.
2	Christiani et al.,	Prospective	447 cotton	37.4	47.7 M	Airborne cotton	Spirometer:	Sex, male,	Cotton workers had small, but
	2001, China	cohort study,	textile work-	(10.6)		dust were	FEV1, FVC	height, age, years	significantly greater, adjusted
		15 years	ers, and 472			measured using		worked,	annual declines in FEV1 than
		follow-up	silk textile			a vertical elutri-		endotoxin	did the silk workers. Years
			workers			ator		exposure,	worked in cotton mills, high
			(control					smokers, change	level of exposure to endotox-
			group)					in FEV1,	in, and across-shift drops in
								respiratory	FEV1 were found to be signifi-
								symptoms	cant determinants for longitu-
									dinal change in FEV1, after
									controlling for appropriate
									confounders. We conclude
									that long-term exposure to
									cotton dust is associated with
									chronic or permanent obstruc-
									tive impairments.

**Table:** 

**Table 2.** The effect of cotton dust exposure on the change in lung function over time, study results (continue)									
**S. No.**	**Author (year) (country)**	**Design, follow-up time**	**Study popu- lation, n**	**Mean age,**	**Sex**	**Cotton dust exposure measurement**	** Outcome: Lung function change measurement **	**Covariates accounted**	**Findings**
3	Wang et al.,	Longitudinal,	447 cotton	56.3	155	Inhalable air	Spirometer:	Age, height,	Cotton workers had more per-
	2005, Shanghai,	20-year fol-	textile; 472	(10.2)	(44.8) M	samplings on	FEV1, FVC	male, smoking,	sistent respiratory symptoms
	China	low-up	silk			airborne cotton		pack-yrs, years	and greater annual declines in
						dust in work		since last	forced expiratory volume in
						area except in		worked, cotton	one second (FEV1) as com-
						final one using		exposure, endo-	pared with silk workers. After
						vertical		toxin level	exposure cessation, in the
						elutriator			final five-yr period, the rate of
									FEV1 decline tended to slow
									in non-smoking males, but
									not in non-smoking females.
									Chronic loss in lung function
									was more strongly associated
									with exposure to endotoxin
									than to dust.
4	Kahraman et al.,	Prospective	196 textile	35.3 ±	37	Airborne cotton	FEV1, FVC,	Gender, smokers	The annual FEV1 loss of cot-
	2013, Türkiye	cohort study,	workers	5.8	(75.5%)	dust were	and PEF	and BMI	ton workers was **53.2 ± 28.4**
		five years fol-			M	measured using	parameter		**mL/year.** Pulmonary function
		low-up				a vertical			parameters of all participants
						elutriator			in 2011 were significantly
									lower than those
									in 2006 (for all, p< 0.05). In
									both surveys, pulmonary func-
									tion in current smokers was
									lower, but this difference was
									not significant (p> 0.05).
5	Shi et al., 2010,	Prospective	447 cotton	60.8 (10)	143	Airborne cotton	FEV1	Age, height,	Years since cessation of tex-
	China	cohort study,	workers and		(45.1) M	dust were		males, smoker,	tilework
		25 years fol-	472 silk			measured using		pack-years, base-	was positively associated with
		low-up	workers from			a vertical elutri-		line FEV1, cessa-	11.3 mL/yr and 5.6 mL/yr in
			Shanghai			ator		tion/retired, years	five-year FEV1 change for cot-
			textile mills					since cessation	ton and silk workers, respec-
								of textile work,	tively. Among male cotton
								annualized	workers, smokers gained more
								changes in FEV1,	FEV1 per year after cessation
								respiratory symp-	of exposure than did non-
								toms	smokers, and the risk of
									symptoms of chronic bronchi-
									tis and byssinosis was larger
									for smoking than for non-
									smoking male cotton workers.

**Table:** 

**Supplement 1.** ???	
** Overview of full electronic search strategy **	**Search strategy**
Lung function	(“lung function” OR “pulmonary function” OR “FEV1” OR “forced expiratory volume” “ OR “spirometry” OR “spirometer” OR “vitalograph”)
Exposures	**AND** (“dust” OR “dusts” OR “cotton dust” OR “cotton” OR “millworkers” OR “mill workers” OR “cotton mill workers” OR “cotton workers” OR “cotton textile workers” OR “textile workers”)
Longitudinal	**AND** (“longitudinal” OR “follow-up” OR “follow up” OR “prospective” OR “cohort”)
Filters	**NOT** “searchword” [Author] **AND** “humans” [MeSH Terms] **AND** English [lang]

mean was used for other surveys. Importantly, the cotton workers had significantly severe annual reduction in FEV1. Among male cotton workers, the decline was 42.3 mL.yr-1 over 20 years while for females it was 24.6 mL.yr-1. The effect of exposure over the course of 20 years was -9.2 mL.yr-1 for male smokers while it was -2.6 mL.yr-1 for male non- smokers and -3.1 mL.yr-1 for female workers, suggesting that there is an additive impact between exposure and smoking (26).

Another study by Christani et al. in 2001, assessed the workers lung function in 15 years follow up study from 1981 to 1996 in China. For cotton employees, the

adjusted annual reductions in FEV1 and FVC were

32.3 mL and 20.1 mL respectively as compared to

29.4 mL and 15.3 mL for silk workers. Male cotton workers’ annual FEV1 decline was 42.2 mL and female employees’ annual decline was 24.8 mL, respectively. Compared to non-smoking silk workers, cotton employees tend to experience larger annual FEV1 reduction. However, the association of cumulative cotton dust exposure was not identified in the workers. The follow-up rates ranged for 70% to 85% for silk workers while 76% to 88% for cotton workers.

A study by Shi et al. showed that yearly FEV1 decline for cotton employees was considerably larger than that for silk workers; however, the rate of decline slowed down over the follow-up period. At the 15, 20, and 25-year follow-up surveys, the annual declines for cotton workers were 232.9 mL/yr, 229.2 mL/yr, and 225.6 mL/yr, respectively. While, for silk workers, the yearly declines were 228.9 mL/yr, 225.0 mL/yr, and -22.5 mL/yr. The study also examined the improvement of lung function after cessation of exposure itself. With respect to the five-year change

in FEV1, there was a positive linear relationship for both cotton and silk employees; however, the rate of improvement was almost twice as rapid for cotton

workers (11.6 vs. 5.6 mL/yr in the two groups, respectively) (19).

Kahraman et al., in 2013, determined the longitudinal variation in lung function among textile workers. The cohort study was initiated in 2006 with 196 cotton textile workers and was ended with 49 textile workers in 2011. Environmental cotton dust was measured using the vertical elutriator while pulmonary function was measured using the standardized methods. Mean age and years of textile workers was 35.3 ± 5.8 years and 7.61 ± 1.83 years

respectively. The yearly decline of FEV1 for all cotton workers was 53.2 ± 28.4 mL. The FEV1 of cotton workers in 2011 were highly lower than to those

done in 2006 (3531.42 ± 711.6 mL versus 3794.48

± 735.4 mL; p< 0.05). Moreover, lung function was lower in current smokers, but this difference was not significant (p> 0.05). Furthermore, as compared to female workers, decline in FEV1 was higher in male workers but it was insignificant. Further, age, height, cumulative dust exposure, pack-years of smoking, and five-year change in BMI were evaluated in relation to five-year losses in pulmonary function, but no significant relationships were identified. The major limitation of the mentioned study was the absence of control group and limited number of cotton textile workers included in respective studies (2).

## DISCUSSION

To our knowledge, this is the first comprehensive review on how long-term exposure to cotton dust affects lung function among cotton textile workers. Overall, this review identified heterogenous findings; and due to small number of studies, we were unable to conduct meta-analyses to assess exposure to

cotton dust causing decrement in FEV1. Three studies showed a significant association between exposure

to cotton dust and change in lung function indices

i.e. FEV1 (17,26,27). The selection of the control group may also have a significant impact on this association. Four studies included control groups within similar jobs but with known lower dust exposures (17,19,26,27). However, one study did not include any control group (2). Therefore, the cotton dust level and its potency may have an impact on the relationship found.

Based on our own independently developed criteria (Table 1), we assigned quality ratings to each included study. All included studies were longitudinal and assessed smoking status as a part of the eligibility criteria. As mentioned in a study, the follow-up time is significant and to assess decline in lung function, longer period of observation is needed to obtain reliable estimates (28) conducted on variation in the flow volume curve with ageing and curve in accordance with the official statement from the American Thoracic Society on spirometry in the occupational settings (29).

The response rate in the included studies differed from 25% to >85%, showing a satisfactory response rate. However, there might be selection bias risk due to variation between responders and non-responders in all the five studies. Since non-responders are the most susceptible individuals, there is a chance that the true impact will be underestimated. However, the reported relationships varied between studies with low and high response rates. In addition, all studies were adjusted for different confounders i.e. smoking, sex, age and height and BMI during analysis phase. However, the confounder selection approach was heterogenic between studies.

It is debatable whether or not baseline lung function should be considered when evaluating changes in lung function over time. Due to correlation problems between lung function level and lung function change, adjusting for the baseline level of lung function is difficult and might result in bias (30). However, we included studies that adjusted for baseline lung function changes as majority of the studies had chosen this approach.

Several cohorts were assessed repeatedly over the follow-up period, and as a result, numerous studies about those cohorts were published. However, we reported studies based on the publications that are most relevant to our interest. The Shanghai textile workers research with five-year gaps between 1981

and 2011, and these cycles produced several findings (12,17,19,26,31-34). They identified no exposure response association but significant differences between cotton and silk workers (17). The Shanghai textile worker study examined the improvement of lung function following cessation of exposure and

witnessed an improvement in FEV1 following cessation of cotton and silk workers (12,34) and

recognised prior occupational exposure levels and sex as significant moderators of FEV1 recovery (34). The Shanghai study (35) also looked at genotypes linked to enzyme activity and revealed that slow enzyme activity genotypes resulted in inefficient metabolising of reactive oxygen species produced by endotoxin exposure, which may ultimately lead to a rapid decrease in lung function. Future research into the biological mechanisms underlying this problem is now possible although that is outside the scope of this review.

The adverse impact of cotton dust on lung function changes are biologically plausible. The inhalation of cotton dust and its deposition in the airway interact with the immune cell which leads to inflammatory response resulting in lung function decline. There is intricate combination between dose and timing of exposure, other environmental conditions, and genetic predisposition determines individuals’ immune response to dust and endotoxins (36). According to the review by Omland et al., in 2014, the mechanisms that relate the immunological response in the lung with potential augmented lung function loss may also involve genetic factors, immune regulation, and mechanisms in relation to cellular repair and the resolution of inflammation (37). Therefore, future review can address this determinant in long-term changes in lung function.

Even though the studies are longitudinal, it cannot be guaranteed that the outcome occurs at the same time as the exposure because the lung function reduction may have occurred earlier in the follow-up period before the exposure was evaluated at a later time. Repeated evaluations of lung function and exposure during follow-ups are one key strategy to deal with this. Although several studies had repeated cycles, analyses were often done at each follow-up period without considering changes in exposure and lung function over the course of the entire follow-up.

The included studies’ mean baseline ages varied from

3. to 60.8 years. The nature of lung function over the course of a lifetime is yet under discussion;

however, age is a significant factor for lung function change. Lung function increases between childhood and adolescence (12). In early adulthood, there is then a plateau phase where FEV1 changes very little or not at all. Afterward this plateau, lung functions begin to decline around 20 years of age. Thus, it is possible to hypothesize that the exposure effect would vary depending on the participants’ ages, but the review doesn’t report this finding. Furthermore, selection bias due to healthy workers is a challenge in most longitudinal studies. Dropouts in the follow- up studies results in the probable selection bias. It is possible that the rate of functional losses among cotton workers might be underestimated based on the dropout, as number of years in the cotton mills and respiratory illness/symptoms were found to be important risk factors for chronic lung function losses. According to a study, average lung function decline over the first year with grain dust exposure was linked with the exposure duration itself, that is a lower reduction in lung function during the first year of exposure predicted a longer subsequent duration of exposure (37). They suggested that the analyses restriction to the “survivors” may underestimate the association between the lung function impairment and dust exposure. Similarly, if the employees had desired to change their job or working place condition, they might have contributed to bias and caused the miss-representation of respiratory symptoms. This might be a glaring issue in every study examined in this review, and it might even imply that the correlations observed are underestimated.

The findings of this review explain the criticality of measuring lung function changes in occupational settings. It is important to focus on the potential negative health impacts linked to occupational exposure to cotton dust in order to protect workers in textile mills with high cotton dust exposure. Future research in this field should focus on maintaining robust follow-up studies, acquiring accurate exposure measurements, and accounting for healthy worker selection bias (i.e., monitoring young cohorts and examining both active workers and dropouts).

## CONCLUSION

The studies included in this review were of different follow-up times with inconsistent findings, and we therefore conclude that there is lack of evidence of a causal relationship between cotton dust exposure and longitudinal excessive lung function decline.

Special attention should be given to pulmonary effects of cotton dust exposure in textile workers to plan appropriate intervention strategies in order to reduce its detrimental effects.

## CONFLICT of INTEREST

The authors declare that they have no conflict of interest.

## AUTHORSHIP CONTRIBUTIONS

Concept/Design: NAA, AK Analysis/Interpretation: NAA, NA Data acqusition: NAA, NA Writing: All of authors

Clinical Revision: All of authors Final Approval: All of authors

## REFERENCES

1. *
  Farokhi A, Heederik D, Smit LA. Respiratory health effects of exposure to low levels of airborne endotoxin-a system- atic review. Environ Health 2018; 17(1): 1-20. https://doi. org/10.1186/s12940-018-0360-7*
2. *
  Kahraman H, Sucakli MH, Kilic T, Celik M, Koksal N, Ekerbicer HC. Longitudinal pulmonary functional loss in cotton textile workers: A 5-year follow-up study. Med Sci Monit 2013; 19: 1176-82. https://doi.org/10.12659/ MSM.889681*
3. *
  Dangi BM, Bhise AR. Cotton dust exposure: Analysis of pulmonary function and respiratory symptoms. Lung India 2017; 34(2): 144-9. https://doi.org/10.4103/0970- 2113.201319*
4. *
  Bolund AC, Miller MR, Sigsgaard T, Schlünssen V. The effect of organic dust exposure on long-term change in lung function: A systematic review and meta-analysis. Occup Environ Med 2017; 74(7): 531-42. https://doi. org/10.1136/oemed-2016-103963*
5. *
  Global Initiative for Asthma (GINA). Global strategy for asthma management and prevention, 2015. Available from: http://www.ginasthma.org/.*
6. *
  Global Initiative for Chronic Obstructive Lung Disease (GOLD). Global strategy for the diagnosis, management and prevention of COPD, 2015. Available from: http:// www.goldcopd.org/.*
7. *
  Knuiman MW, James AL, Divitini ML, Ryan G, Bartholomew HC, Musk A. Lung function, respiratory symptoms, and mortality: Results from the Busselton Health Study. Ann Epidemiol 1999; 9(5): 297-306. https://doi.org/10.1016/ S1047-2797(98)00066-0*
8. *
  Schu HJ, Dorn J, Grant BJ, Winkelstein Jr W, Trevisan M. Pulmonary function is a long-term predictor of mortality in the general population: 29-year follow-up of the Buffalo Health Study. Chest 2000; 118(3): 656-64. https://doi. org/10.1378/chest.118.3.656*
9. *
  Hole D, Watt G, Davey-Smith G, Hart C, Gillis C, Hawthorne V. Impaired lung function and mortality risk in men and women: Findings from the Renfrew and Paisley prospective population study. BMJ 1996; 313(7059): 711- 5. https://doi.org/10.1136/bmj.313.7059.711*
10. *
  Singh-Manoux A, Dugravot A, Kauffmann F, Elbaz A, Ankri J, Nabi H, et al. Association of lung function with physical, mental and cognitive function in early old age. Age 2011; 33(3): 385-92. https://doi.org/10.1007/s11357-010- 9189-x*
11. *
  Henderson A. Chronic respiratory failure. Practitioner 1994; 238(1538): 345-50.
  *
12. *
  Shi J, Mehta AJ, Hang JQ, Zhang H, Dai H, Su L, et al. Chronic lung function decline in cotton textile workers: Roles of historical and recent exposures to endotoxin. Environ Health Perspect 2010; 118(11): 1620-4. https:// doi.org/10.1289/ehp.0901178*
13. *
  Wang XR, Pan LD, Zhang HX, Sun BX, Dai Hl, Christiani DC. Follow‐up study of respiratory health of newly‐hired female cotton textile workers. Am J Ind Med 2002; 41(2): 111-8. https://doi.org/10.1002/ajim.10042*
14. *
  Wang X, Eisen E, Zhang H, Sun B, Dai H, Pan L, et al. Respiratory symptoms and cotton dust exposure; results of a 15 year follow up observation. Occup Environ Med 2003; 60(12): 935-41. https://doi.org/10.1136/ oem.60.12.935*
15. *
  Bossé R, Sparrow D, Garvey AJ, Costa Jr PT, Weiss ST, Rowe JW. Cigarette smoking, aging and decline in pulmo- nary function: A longitudinal study. Arch Environ Health 1980; 35(4): 247-52. https://doi.org/10.1080/00039896*

*.1980.10667500*

16. *
  Su YM, Su JR, Sheu JY, Loh CH, Liou SH. Additive effect of smoking and cotton dust exposure on respiratory symp- toms and pulmonary function of cotton textile workers. Ind Health 2003; 41(2): 109-15. https://doi.org/10.2486/ indhealth.41.109*
17. *
  Wang X, Zhang HX, Sun BX, Dai HL, Hang JQ, Eisen E, et al. Cross-shift airway responses and long-term decline in
  *

*
FEV1 in cotton textile workers. Am J Respir Crit Care Med 2008; 177(3): 316-20. https://doi.org/10.1164/*

*rccm.200702-318OC*

18. *
  Cooper J. Occupational asthma, byssinosis, and industrial bronchitis. Fishman’s pulmonary diseases and disorders McGraw-Hill, New York, NY. 1998: 915-24.
  *
19. *
  Christiani DC, Wang XR, Pan LD, Zhang HX, Sun BX, Dai H, et al. Longitudinal changes in pulmonary function and respiratory symptoms in cotton textile workers. A 15-yr follow-up study. Am J Respir Crit Care Med 2001; 163(4): 847-53. https://doi.org/10.1164/ajrccm.163.4.2006063*
20. *
  Liebers V, Brüning T, Raulf‐Heimsoth M. Occupational endotoxin‐exposure and possible health effects on humans. Am J Ind Med 2006; 49(6): 474-91. https://doi. org/10.1002/ajim.20310*
21. *
  Daba Wami S, Chercos DH, Dessie A, Gizaw Z, Getachew A, Hambisa T, et al. Cotton dust exposure and self-report- ed respiratory symptoms among textile factory workers in Northwest Ethiopia: A comparative cross-sectional study. J Occup Med Toxicol 2018; 13: 13. https://doi. org/10.1186/s12995-018-0194-9*
22. *
  Arksey H, O’Malley L. Scoping studies: Towards a meth- odological framework. Int J Soc Res Methodol 2005; 8(1): 19-32. https://doi.org/10.1080/1364557032000119616*
23. *
  Levac D, Colquhoun H, O’Brien KK. Scoping studies: Advancing the methodology. Implement Sci 2010; 5: 69. https://doi.org/10.1186/1748-5908-5-69*
24. *
  Jüni P, Witschi A, Bloch R, Egger M. The hazards of scor- ing the quality of clinical trials for meta-analysis. JAMA 1999; 282(11): 1054-60. https://doi.org/10.1001/ jama.282.11.1054*
25. *
  Stroup DF, Berlin JA, Morton SC, Olkin I, Williamson GD, Rennie D, et al. Meta-analysis of observational studies in epidemiology: A proposal for reporting. JAMA 2000; 283(15): 2008-12. https://doi.org/10.1001/ jama.283.15.2008*
26. *
  Wang X, Zhang H, Sun B, Dai H, Hang J, Eisen E, et al. A 20-year follow-up study on chronic respiratory effects of exposure to cotton dust. Eur Respir J 2005; 26(5): 881-6. https://doi.org/10.1183/09031936.05.00125604*
27. *
  Shi J, Hang JQ, Mehta AJ, Zhang HX, Dai Hl, Su L, et al. Long-term effects of work cessation on respiratory health of textile workers: A 25-year follow-up study. Am J Respir Crit Care Med 2010; 182(2): 200-6. https://doi. org/10.1164/rccm.200903-0329OC*
28. *
  Burrows B, Lebowitz MD, Camilli AE, Knudson RJ. Longitudinal changes in forced expiratory volume in one second in adults: Methodologic considerations and find- ings in healthy nonsmokers. Am Rev Respir Dis 1986; 133(6): 974-80.
  *
29. *
  Redlich C, Tarlo S, Hankinson J, Townsend M, Eschenbacher W, Von Essen S, et al. American Thoracic Society Committee on Spirometry in the Occupational Setting. Official American Thoracic Society technical standards: Spirometry in the occupational setting. Am J Respir Crit Care Med 2014; 189(8): 983-93. https://doi.org/10.1164/ rccm.201402-0337ST*
30. *
  Irwig L, Groeneveld H, Becklake M. Relationship of lung function loss to level of initial function: Correcting for measurement error using the reliability coefficient. J Epidemiol Community Health 1988; 42(4): 383-9. https:// doi.org/10.1136/jech.42.4.383*
31. *
  Christiani DC, Ye Tt, Zhang S, Wegman DH, Eisen EA, Ryan LA, et al. Cotton dust and endotoxin exposure and long‐term decline in lung function: Results of a longitudi- nal study. Am J Ind Med 1999; 35(4): 321-31. https://doi. org/10.1002/(SICI)1097-0274(199904)35:4<321::AID- AJIM1>3.0.CO;2-L
  *
32. *
  Wang XR, Zhang HX, Sun BX, Dai HL, Pan LD, Eisen EA, et al. Is chronic airway obstruction from cotton dust expo- sure reversible? Epidemiology 2004; 15(6): 695-701. https://doi.org/10.1097/01.ede.0000142141.28139.25*
33. *
  Lai PS, Hang JQ, Zhang FY, Lin X, Zheng BY, Dai HL, et al. Gender differences in the effect of occupational endotoxin exposure on impaired lung function and death: The Shanghai Textile Worker Study. Occup Environ Med 2014; 71(2): 118-25. https://doi.org/10.1136/oemed-2013- 101676*
34. *
  Lai PS, Hang JQ, Valeri L, Zhang FY, Zheng BY, Mehta AJ, et al. Endotoxin and gender modify lung function recovery after occupational organic dust exposure: A 30-year study. Occup Environ Med 2015; 72(8): 546-52. https://doi. org/10.1136/oemed-2014-102579*
35. *
  Hang J, Zhou W, Wang X, Zhang H, Sun B, Dai H, et al. Microsomal epoxide hydrolase, endotoxin, and lung func- tion decline in cotton textile workers. Am J Respir Crit Care Med 2005; 171(2): 165-70. https://doi.org/10.1164/ rccm.200407-888OC*
36. *
  Liebers V, Raulf-Heimsoth M, Brüning T. Health effects due to endotoxin inhalation. Arch Toxicol 2008; 82(4): 203-10. https://doi.org/10.1007/s00204-008-0290-1*
37. *
  Omland Ø, Würtz ET, Aasen TB, Blanc P, Brisman J, Miller MR, et al. Occupational chronic obstructive pulmonary disease: A systematic literature review. Scand J Work Environ Health 2014: 19-35. https://doi.org/10.5271/ sjweh.3400*

